# The Psychological Effect of Forming WeChat Groups Between Medical Staff and Patients With COVID-19

**DOI:** 10.3389/fpubh.2021.586465

**Published:** 2021-06-23

**Authors:** Li-Ping Yuan, Zheng-Hao Yu, Xian-Cui Zhang, Wei Zhang, Ling-Li Jin, Zhen Wang, Jin-Sun Yang, Hou-Bao Huang, Qu Zhang, Xiu-Bin Tao

**Affiliations:** Department of Intensive Care Unit, Yijishan Hospital of Wannan Medical College, Wuhu, China

**Keywords:** 2019-nCoV, pneumonia, disease response, psychological care, health education, satisfaction, WeChat group

## Abstract

**Background:** This study was conducted in order to explore the effect of psychological intervention based on the use of WeChat with coronavirus disease 2019 (COVID-19) patients.

**Methods:** A total of 65 patients with COVID-19, from two wards, were divided into an experimental group and a control group with the ward as the basic unit. Communication concerning routine treatment and nursing was established between the medical staff and patients in the experimental group *via* WeChat groups. Within 48 h of admission, at 7 days, and on discharge, all 65 patients completed two self-evaluation questionnaires: the Positive and Negative Affect Schedule (PANAS) and the Hospital Anxiety and Depression Scale (HADS). Hospital stay statistics and a satisfaction survey on discharge were also collated for both groups of patients.

**Results:** The PANAS scores of the experimental group were 26.61 ± 7.99 points on admission, 20.81 ± 5.48 points at 7 days, and 19.58 ± 6.61 points on discharge (*P* < 0.05). The scores of HADS in the experimental group were 27.74 ± 9.35 points on admission, 12.19 ± 1.92 points at 7 days, and 11.71 ± 3.64 points on discharge (*P* < 0.05). The differences in the PANS and HADS scores between the experimental and control groups at 7 days and on discharge were statistically significant. The discharge satisfaction ratings of the two groups of patients were 99.87 ± 0.34 and 98.68 ± 1.09 points, the difference being statistically significant (*t* = 5.827, *P* < 0.05).

**Conclusion:** Establishing WeChat groups between medical staff and patients with COVID-19 and building a bridge for better communication improved patients' positive mentality and their compliance with doctors, shortened their hospital stay, and promoted their recovery.

## Background

Coronavirus disease 2019 (COVID-19) is a new type of acute infectious disease caused by infection with the 2019 novel coronavirus (2019-nCoV). It has a high level of infectivity and a relatively high mortality rate (1). China has placed the disease in the category of class B infectious diseases, but prevention and control measures (2, 3) for class A infectious diseases are necessary. Patients with COVID-19 are hospitalized in isolation, and their family members cannot accompany or visit them. Medical staff wear protective suits, goggles, or face masks, which affect the normal communication between medical staff and patients. In order to avoid infection and cross-infection, patients are confined to the wards as much as possible, and at the same time, the sudden stressful event of COVID-19 has a huge impact on the daily lives and the physical and mental health of their families and the public in general. These changes can cause psychological problems for patients, including tension, anxiety, depression, fear, and even despair (3, 4). In response to the current situation, our provincial medical team supporting Wuhan established a WeChat group between medical staff and patients to conduct health education and lung rehabilitation training guidance (5–9), address items missed during ward rounds, track medication effects, and promptly answer patients' questions and doubts.

## Materials and Methods

### General Information

Patients with COVID-19 admitted to a hospital in Wuhan between February 2020 and March 2020 were selected as the research participants. The inclusion criteria were as follows: (1) ability to read and understand the Chinese language, (2) age above 14 years, (3) no significant organ dysfunction, (4) no mental disorder, and (5) willingness to join a WeChat group. The exclusion criteria were as follows: (1) a history of mental illness or inability to communicate and (2) a hospital stay of <48 h. Of the two wards taken over by the treatment team, one was designated the experimental group with a total of 31 patients, 15 male and 16 female, aged 28–88 years, and the other ward was designated the control group with 34 patients, 20 male and 14 female, aged 25–79 years. The educational background of the two groups of patients was elementary school and above, and they were all able to communicate normally with the medical staff.

### Methods

This study used an experimental design where the researchers aimed to test the effectiveness of an intervention. The patients in the control group received routine treatment and nursing. They were seen by doctors twice a day, and if they had any problems or needs, they could call the nurses for help. The patients in the experimental group joined a WeChat group. After they were admitted to the hospital, they could scan the code to join the WeChat group voluntarily. The treatment team leader, each nursing team leader and the psychotherapist, respiratory and rehabilitation therapist, and other personnel joined the WeChat group, and the family members of the elderly patients were invited to join as well. The head nurse was the group manager, and other medical staff could voluntarily join the WeChat group. The physicians told patients the results of examinations and tests every afternoon, answered their patients' doubts about the disease at any time, and informed them of the effects of their treatment. When patients showed anxiety about the disease or missed their families, the nursing staff sent them texts and photos or made voice or video calls to them to provide them with psychological comfort, too. The nurse on duty checked the information in the group on the ward mobile phone WeChat and provided timely psychological care or psychological intervention with the help of the psychotherapist at the patient's bedside. Patients in the group shared recovery stories and family members in the group comforted and encouraged patients and, thus, helped them to build confidence about overcoming the disease. In addition, health education information about the novel coronavirus from various information platforms was passed on to patients through the WeChat groups. Patients received videos, written materials, and other related self-management information (10), thus, making it possible for them to be given targeted medication guidance, diet guidance, and psychological counseling. Every day through WeChat, rehabilitation therapists and respiratory therapists shared lung rehabilitation exercises and respiratory function exercise videos with the group and urged patients to do the exercises. However, information not related to disease or health, such as advertisements, could not be sent to the group. The group could also be used to inform those responsible of patients' dietary requirements, such as the need for more rice, or a preference for porridge or noodles, and special reservations were made every day as required. When elderly patients ran out of their daily necessities, such as toilet paper or nursing pads, they could inform the medical staff or family members so their supplies could be renewed. This all meant that the daily problems and needs of patients were solved and met as far as possible. Patients and medical staff were often sent pictures of the current spring scenery and beautiful landscapes and music to soothe them.

The Positive and Negative Affect Schedule (PANAS) and the Hospital Anxiety and Depression Scale (HADS) questionnaires were completed within 24 h of admission and on discharge. The self-care ability (ADL) assessment was also completed within 24 h of a patient's admission, and a satisfaction survey was conducted upon discharge. Elderly patients were assisted by a nurse if they could not fill in the evaluation forms on their own.

### Observation Indicators

The Medical Coping Modes Questionnaire was designed by H. Feifel. It is a tool used to measure a patient's coping style. It was translated into Chinese by Jiang Qianjin et al. and revised as 20 items, which can test the three dimensions of confrontation, avoidance, and acceptance-resignation. The corresponding questions are as follows: questions 1, 2, 5, 10, 12, 15, 16, and 19 refer to confrontation; questions 3, 7, 8, 9, 11, 14, and 17 refer to avoidance; and questions 4, 6, 13, 18, and 20 refer to acceptance-resignation. Each item is scored on a four-point scale.

The PANAS has a total of 20 items. Questions 1, 3, 5, 9, 10, 12, 14, 16, 17, and 19 refer to positive emotions, and the larger the value, the more positive the emotion. Questions 2, 4, 6, 7, 8, 11, 13, 15, 18, and 20 refer to negative emotions, and the larger the value, the more negative the emotion.

The HADS has two subscales for anxiety and depression, with seven questions addressing anxiety (A) and seven more addressing depression (D). The scores of the anxiety and depression subscales are rated as follows: 0–7 points mean “asymptomatic,” 8–10 points mean “suspicious,” and 11–21 points mean “definitely present.” When scoring, eight is the starting point. That is to say, both “suspicious” and “symptomatic” are positive.

With respect to the patients' hospital stay and their satisfaction, their electronic medical records were consulted and recorded on discharge, and the hospitalization satisfaction questionnaire was activated in the WeChat group or on the WeChat public platform, and the patient or family members filled it out. The maximum score was 100 points.

### Statistical Analysis

SPSS 19.0 statistical software was used for statistical analysis of the data. The measurement data were expressed as mean ± standard deviation ($\bar{X}$ ± s). A *t* test was used for statistical analysis, and the count data were analyzed by a χ^2^ test. The test level was α = 0.05.

## Results

### General Information

There were no statistically significant differences in gender, age, self-care ability (ADL) scores, or other general data between the two groups of patients (*P* > 0.05), as shown in Table 1.

**Table 1:** Comparison of general conditions of two groups of patients.

**Group**	**Cases**	**Gender (Man/Woman)**	**Age**	**ADL score**
Experimental group	31	14/17	56.77 ± 17.01	77.9 ± 20.27
Control group	34	8/9	55.91 ± 16.44	73.65 ± 20.7
χ^2^/*t* value		0.023	0.208	0.836
*P* value		0.087	0.836	0.406

### Comparison of Patients' Disease Coping Styles

There was no statistically significant difference in disease coping styles between the two groups (*P* > 0.05), as shown in Table 2.

**Table 2:** Comparison of disease coping styles between the two groups.

**Group**	**Cases**	**Total score**	**Front**	**Avoid**	**Surrender**
Experimental group	31	49.97 ± 2.33	19.26 ± 2.07	17.29 ± 0.90	13.48 ± 0.72
Control group	34	49.18 ± 2.18	18.47 ± 1.93	17.38 ± 1.81	13.18 ± 1.47
*T* value		1.184	1.594	−0.256	1.055
*P* value		0.241	0.177	0.799	0.295

### Comparison of PANAS Scores

The results of this study showed that there was no statistically significant difference in the PANAS score between the two groups of patients on admission. There was no significant decrease in the PANAS score of the control group on discharge, and the difference was not statistically significant (*P* > 0.05) (see Table 3).

**Table 3:** Comparison of Panas scores between the two groups.

**Category**	**Experimental group (*n* = 31)**	**Control group (*n* = 34)**	***t* value**	***P* value**
Admission (Score)	26.61 ± 7.99	26.26 ± 6.69	0.191	0.849
Discharge (Score)	19.58 ± 6.61	24.53 ± 7.44	−2.824	0.006
Score difference (d ± sd,Score)	7.03 ± 8.49	1.74 ± 9.25	—	—
*t* value	4.613	1.094		
*P* value	0	0.282		

### Comparison of Scores for the HADS

The results of this study showed that the HADS scores of the two groups were not statistically different on admission (*P* > 0.05). However, the HADS scores of the experimental group on discharge were lower than those on admission, and they were significantly lower than those of the control group (see Table 4).

**Table 4:** Comparison of hads score between the two groups.

**Category**	**Experimental group (*n* = 31)**	**Control group (*n* = 34)**	***t* value**	***P* value**
Admission (Score)	14.42 ± 5.24	16.03 ± 3.09	−1.525	0.132
Discharge (Score)	11.71 ± 3.64	15.44 ± 3.86	−4.001	0
Score difference (d ± sd,Score)	2.68 ± 3.98	0.53 ± 2.55	—	—
*t* value	3.747	1.211		
*P* value	0.001	0.235		

### Comparison of Hospital Stay and Patient Satisfaction

The results of this study showed that there was no statistical difference in the length of hospitalization between the two groups of patients. The hospitalization satisfaction of the patients in the experimental group was higher than that in the control group, and the difference was statistically significant, as shown in Table 5.

**Table 5:** Comparison of hospital stay and satisfaction between the two groups.

**Group**	**Cases**	**Hospital stay**	**Satisfaction**
Experimental group	31	15.52 ± 7.25	99.87 ± 0.34
Control group	34	15.09 ± 6.89	97.14 ± 1.59
*t* value		0.244	8.637
*P* value		0.808	0

## Discussion

Individuals are prone to exaggerated thinking when facing emergencies (11). When it comes to COVID-19, a new type of acute infectious disease, patients suffering from it are likely to experience fear. Once patients are admitted to the hospital, their activity space is narrow and confined, and they are cut off from families and friends. They may consequently experience mood swings and various negative emotions, such as loneliness, and they may miss loved ones and exhibit childish behavior (12, 13), so they may urgently seek social support in order to relieve their emotions (12). Through the intervention of the WeChat group, the mentality of the patients in the experimental group became positive. Every day when the head nurse did the rounds, the patients in the experimental group were positive and enthusiastic, carrying out indoor activities, and they were able to communicate normally. In contrast, the patients in the control group were anxious, lying in bed, and talking about the results of the laboratory tests and their own progress.

Thick protective suits can also hinder effective communication between the medical staff and patients, but with the establishment of the WeChat group, its members were able to interact and communicate with ease anytime and anywhere. The medical staff could provide patients with information about their disease (14), which enabled the patients to better understand their situation and, thus, feel more confident in their treatment. This was also a form of psychological care, narrowing the distance between medical staff and patients, building a sense of trust, and bringing more hope and warmth to the patients. It would seem that health education and psychological counseling can eliminate patients' negative emotions (15) and make them more positive and optimistic.

Studies have shown that providing patients with health education through the WeChat platform can improve their compliance with doctors. By providing disease-related knowledge to WeChat groups and responding to patients' doubts, patient compliance with medication is also improved (16, 17) and they are more willing to follow the doctor's instructions and cooperate with treatment. After the daily rounds, doctors adjust the treatment as appropriate, adding infusion therapy or changing the infusion therapy to oral medication, for example. The physician can keep patients informed *via* the WeChat group and explain the reasons behind their decisions, and as a result, patients are aware of the effects of the treatment and the next stage of the treatment plan. When patients comply with their doctors, they also gain the confidence to fight against the disease, and when compliance is 100%, the treatment plan is more effective in promoting recovery.

The WeChat group members in our study also helped to create a positive atmosphere by posting some beautiful pictures of the spring outside, and the mutual encouragement among patients also created a positive atmosphere. Some researchers used the WeChat platform to carry out nursing interventions for patients with malignant tumors, which effectively relieved patients' pain and also improved their medication compliance (18). There was a patient on the ward who remained positive after the fifth nucleic acid test and felt extremely anxious and depressed. Other patients in the group informed her that some patients in another hospital had had the same experience and did not become negative until the seventh test and that there was even a patient who remained positive even after the seventh test. On hearing this, the patient was relieved to find out that she was not alone. As it was still possible for her tests to turn negative, her mood improved significantly, and she actively cooperated with her treatment and examinations. Therefore, peer education and encouragement appear to help ensure the effective treatment of patients.

Establishing WeChat groups in hospitals can also improve satisfaction and promote patient recovery. The number of users and range of coverage of WeChat nowadays is very large. In 2018, the average daily login user number of WeChat was 1.01 billion according to its official website, and 45 billion WeChat messages were sent every day (19). More and more hospitals have begun to use WeChat as a form of health education and continuing care. WeChat has become the main platform for health communication (20). Studies have shown that health education through WeChat groups can promote patients' understanding of their disease and improve their self-management capabilities, thereby greatly promoting patient recovery (21). In the WeChat group, medical staff could explain self-care methods with regard to the novel coronavirus disease and convey related knowledge of how to avoid cross-infection and infection, teach patients the appropriate rehabilitation exercises, and impart protection knowledge, and patients could encourage and give guidance to each other. All of this helped to increase patients' confidence in their chances of beating the disease and their compliance with treatment. A positive mood also helped shorten patients' hospital stay, promote recovery, and improve their quality of life, which resulted in improved nursing satisfaction (22, 23). Although there was no significant difference in the length of hospitalization between the two groups of patients in this study, this may be related to the long recovery time of COVID-19 and the unified deployment of admission and discharge for all patients by the Wuhan City headquarters.

## Conclusion

In summary, the application of a WeChat group among patients with COVID-19 proves to have had positive psychological effects on patients, especially as a means of providing a communication hub in the face of a significant public health emergency. This success cannot be ignored, and the use of WeChat groups is worthy of reference and promotion in clinical practice.

## Data Availability Statement

The raw data supporting the conclusions of this article will be made available by the authors, without undue reservation.

## Ethics Statement

The studies involving human participants were reviewed and approved by Yijishan Hospital of Wannan Medical College. The ethics committee waived the requirement of written informed consent for participation.

## Author Contributions

L-PY and Z-HY were responsible for the concept of the study. X-CZ, WZ, L-LJ, ZW, J-SY, H-BH, QZ, and X-BT participated in the design and coordination of the study. X-BT helped to draft the manuscript. All authors read and approved the final version of the manuscript.

## Conflict of Interest

The authors declare that the research was conducted in the absence of any commercial or financial relationships that could be construed as a potential conflict of interest.

## Abbreviations

ICU: intensive care unit
PANAS: Positive and Negative Emotion Scale
HADS: Anxiety and Depression Scale.
